# Ligand-independent EphB1 signaling mediates TGF-β-activated CDH2 and promotes lung cancer cell invasion and migration

**DOI:** 10.7150/jca.44576

**Published:** 2020-04-07

**Authors:** Lujuan Wang, Qiu Peng, Buqing Sai, Leliang Zheng, Jiaqi Xu, Na Yin, Xiang Feng, Juanjuan Xiang

**Affiliations:** 1 Hunan Cancer Hospital, the Affiliated Cancer Hospital of Xiangya School of Medicine, Central South University, Changsha, Hunan, PR China; 2 Cancer Research Institute, School of Basic Medical Science, Central South University, Changsha, Hunan, China; 3 NHC Key Laboratory of Carcinogenesis and the Key Laboratory of Carcinogenesis and Cancer Invasion of the Chinese Ministry of Education, Xiangya Hospital, Central South University, Changsha, Hunan, China; 4 Hunan Key Laboratory of Nonresolving Inflammation and Cancer, Changsha, Hunan, 410013, China; 5 Department of Oncology, The Second Xiangya Hospital, Central South University, Changsha, Hunan 410011, P.R. China

**Keywords:** EphB1, TGF-β, lung cancer, metastasis

## Abstract

**Purpose**: The initial step of cancer metastasis is that cancer cells acquire the capability to migrate and invade. Eph receptors comprise the largest family of receptor tyrosine and display dual role in tumor progression due to unique ephrin cis- or trans- signaling. The roles of EphB1 and its phosphorylation signaling in lung cancer remain to be elucidated.

**Patients and Methods**: We analyzed the expression of EphB1 in both publicly available database and 60 cases of NSCLC patients with or without metastasis. The migration and invasion of lung cancer cells were assessed by a transwell assay. The activation of EphB1 signaling was assessed by western blot and real-time PCR. The EphB1 mutant was used to evaluate the effect of phosphorylation of EphB1.

**Results**: Here, we showed that increased expression of EphB1 was detected in Non-Small-Cell Lung Cancer (NSCLC) biopies compared to non-cancer controls. Significant higher expression of EphB1 in lung biopsies were found in patients with metastasis compared to non-metastatic NSCLC patients. Higher EphB1 expression was correlated with poor patient survival in lung cancer. Overexpression of EphB1 promoted the migration and invasion of lung cancer cells. On the contrast, Ephrin-B2, a transmembrane ligand for EphB1 forward signaling, inhibited migration and invasion of lung cancer cells. TGF-β-activated Smad2 transcriptionally upregulated the endogenous expression of EphB1. Ligand-independent EphB1 promoted Epithelial-mesenchymal transition (EMT) through upregulating CDH2.

**Conclusion**: Our results showed that the effect of EphB1 on the migration and invasion was context-specific and was dependent on EphB1 phosphorylation.

## Introduction

Non-small cell lung cancer (NSCLC) accounts for approximately 80% of lung cancer cases. Metastasis is the major reason for the mortality of lung cancer patients. Cancer cell migration and invasion are initial steps in metastasis^1^,^2^. Epithelial-mesenchymal transition (EMT) of cancer cells is believed to be crucial for cancer cell invasion^3^. The process that epithelial cancer cells lose their polarity and displays mesenchymal phenotype is called EMT. Eph receptors, which comprise the largest family of receptor tyrosine kinase, have been found to play a role in EMT^4^. The Eph receptors are divided into 2 subclasses: nine EphA receptors and five EphB receptors. Ephs and their ligands ephrins trigger bidirectional signal pathway upon cell-to-cell contact^5^. Eph/ephrin interactions have been implicated in various pathologic processes, including inflammation, neural development, and angiogenesis^6^-^8^. Eph forward signaling that depends on Eph kinase activity has been involved in cell migration and evasion^5^. However, bidirectional signals can also mediate cell repulsion^5^.

Eph receptors display dual role in tumor progression and tumor suppression^9^. EphB subgroup and the Ephrin-B subgroup are coexpressed in SCLC cell lines and tumors, modulating the behavior of SCLC through autocrine or juxtacrine activation^10^. EphA/B mutation or amplification can be found in 16% of lung adenocarcinoma patients^11^. EphB3 promotes cancer cell survival and migration by enhancing DNA synthesis and inhibiting apoptosis in NSCLC cells^12^. Eph receptors that are activated by ephrins can inhibit oncogenic signaling pathways, such as the HRAS-Erk, PI3K-Akt and Abl-Crk pathways^5^. The paradox may be because of the cis and trans signaling or ligand-dependent or ligand-independent Eph signaling.

In this study, we found that ligand-independent EphB1 promoted lung cancer cell mobility and invasion. TGF-β-activated Smad2 transcriptionally interacted with a Smad2-binding element in EphB1. Ligand-independent EphB1 promotes EMT through upregulating CDH2. However, the ligand induced EphB1 phosphorylation inhibited lung cancer mobility and invasion. A better understanding of context of EphB1 signaling can help to explain the paradox roles in cancer progression.

## Materials and Methods

### Antibodies and Reagents

Antibodies to proteins were obtained from the following sources: EphB1 (#ab129103) and phos-EphB1 (#ab61791) for Western blot were purchased from Abcam; EphB1 (#AF542) for immunohistochemistry was purchased from Novus Biologicals; N-cadherin (#13116) and E-cadherin (#3195) were from Cell Signaling Technology; β-actin (#60008-1-Ig), Smad2 (#12570-1-AP) and GAPDH (#60004-1-Ig) were from Proteintech. Recombinant human Ephrin-B2 Fc chimera protein was purchased from R&D (7397-EB, RD Inc, MN, USA) and Recombinant Human TGF-β1 (#CA59) was purchased from Novoprotein.

### Cell culture, plasmid construction, siRNA and patients

NSCLC cell lines A549 and H460 were cultured in RPMI-1640 medium supplemented with penicillin G (100 U/mL), streptomycin (100 mg/mL) and 10% fetal calf serum. HEK293 cells were cultured in Dulbecco's modified Eagle medium (Gibco) with 1 g/L glucose and 10% FBS. All cell lines were obtained from ATCC. Cells were grown at 37°C in a humidified atmosphere of 5% CO_2_ and were routinely sub-cultured using 0.25% (w/v) trypsin-EDTA solution.

DNA fragments encoding Flag-Smad2, GFP-EphB1 were generated by PCR and cloned into a Flag-tagged (p3xFLAG-CMV-10) or GFP-tagged (pEGFP-N1) empty vector. Mutant versions of the EphB1 Y594 region was generated using site-directed mutagenesis with the Vazyme Mut Express™ Fast Mutagenesis Kit. The vectors were denoted as EphB1-Y594-mutant. The primers used to construct plasmids are as follows: 5'-GATGAAGATCTGCATTGACCCCTTCACTTACGAGGATCCC-3'; 5'-AGGGGTCAATGCAGATCTTCATCCCTGGGGAGCCTCGGCC-3'. si-EphB1: 5'-GTCCCATGAAAAGACTTAA-3', si-Smad2: 5′-AGACGTCCATCATTCTGGA-3′ negative control (NC) siRNA duplexes were purchased from Ribobio (Guangzhou, China). Plasmids and siRNAs were transfected into cells using Lipofectamine 3000 (Invitrogen).

Patients diagnosed with NSCLC (n=60) were included in this study, 13 of which were diagnosed with lymph node-positive lung cancer and 47 of which were diagnosed with lymph node-negative lung cancer. All cases enrolled in this study were diagnosed at the second Xiangya hospital, Central South University, China. The clinical characteristics of the cases are summarized in Table 1. The patients were informed of the sample collection and signed informed consent forms. The collection and use of samples were approved by the ethical review committees of the second Xiangya Hospital, Central South University. Clinicopathological characteristics of these patients are presented in Table 1.

### Western blotting

The protein lysate used for western blotting was extracted using RIPA buffer (Biotime, Hangzhou, China) containing protease inhibitors (Roche, Basel, Switzerland). Proteins were quantified using the BCA^TM^ Protein Assay Kit (Pierce, USA). A western blot system was set up using a Bio-Rad Bis-Tris Gel system, according to the manufacturer's instructions (Bio-Rad, CA, USA). The cell protein lysates were separated on 10% SDS-polyacrylamidegels and electrophoretically transferred to polyvinylidene difluoride membranes (Millipore, Danvers, MA, USA). The primary antibody solution was prepared in 5% blocking buffer. Primary antibodies against EphB1 (Abcam, USA), p-EphB1 (Abcam, USA) were incubated with the membrane at 4 ºC overnight, followed by a brief wash and incubation with secondary antibody for 1 h at room temperature. An anti-GAPDH antibody control was purchased from Proteintech (Chicago, USA) and was used as a loading control. Finally, a 40:1 solution of peroxide and luminol was added to cover the blot surface for five minutes at room temperature. The chemiluminescent signals were captured, and the intensity of the bands was quantified using a Bio-Rad ChemiDoc XRS system (Bio-Rad, CA,USA).

### Cell migration and invasion assays

Cell migration and invasion assays were both performed using a transwell insert that contains polycarbonate filters with 8-μm pores (cat. no. 3422; Corning). Cells (5x10^4^) were suspended in 200 µl of serum-free medium and added to the transwell membrane in the upper chamber. Migrated cells were fixed in 4% paraformaldehyde and stained with crystal violet. Migrated cell images were observed and imaged under microscope (CKX41, Olympus, Japan). Cell migration was quantitated by counting in 10 random fields on the lower membrane surface. Invasion capacity of cells was measured by Matrigel matrix gel invasion assay. The surface of the filter (8-μm pore size) of the upper chamber was coated with 1 mg/ml Matrigel matrix. Cells (5x10^4^) were suspended in 200 µl of serum-free medium and added to the transwell membrane in the upper chamber. Invaded cells were fixed in 4% paraformaldehyde and stained with crystal violet. Cell invasion was observed and imaged under microscope (CKX41, Olympus, Japan). Cell invasion was quantitated by counting in 10 random fields on the lower membrane surface.

### Immunohistochemistry

Lung biopsies were fixed and embedded in paraffin wax. Four- to six-μm thick paraffin sections were defaced followed by hydration. Tissue sections were incubated with primary antibody at 4°C overnight in a humidified chamber. After extensive washing with PBS, sections were incubated with biotin-linked goat anti-rabbit IgG antibodies (UltraSensitive S-P Kit, Maixin Biotechnology Company, Fuzhou, China). The sections were then washed and followed by developing in 3'-diaminobenzidine hydrochloride (DAB) as chromogen, and sections were counterstained with haematoxylin. Finally, after dehydration and mounting, the sections were observed and imaged under microscope (OLYMPUS BX-51, Japan). Goat serum and PBS were used instead of the first antibody as a negative control and blank control respectively. A semi-quantitative scoring criterion for IHC was used in which both the staining intensity and positive areas were recorded.

### Quantitative real-time PCR

Total RNA for RNA-seq experiment was used for real-time PCR to confirm the expression of genes. cDNA was synthesized from total RNA using the RevertAid First Strand cDNA Synthesis Kit (Thermo Scientific, Waltham, MA, USA). GAPDH was used as the endogenous control. Quantitation PCR was performed according to the indications. Real-time PCR was performed using the Bio-Rad IQ^TM5^ Multicolor real-time PCR detection System (Bio-Rad, Berkeley, CA, USA). Relative mRNA expression levels were calculated by the 2^-ΔΔCt^ method. The siRNA sequences for knockdown of target genes are shown in Table 2.

### Bioinformatics analysis

Six independent cohorts of lung cancer data and their correlated clinic data, GSE10072, GSE19188, GSE7670, GSE68465^13^, GSE50081^14^ and GSE30219^15^ were collected from the National Center for Biotechnology Information's Gene Expression Omnibus (GEO, NCBI). GSE10072 included 58 lung cancer samples and 49 normal lung samples; GSE19188 included 45 lung cancer samples and 65 normal lung samples; GSE7670 included 27 lung cancer samples and 30 normal lung samples; GSE68465 included 443 lung cancer samples and 19 normal lung samples; GSE50081 included 293 lung cancer samples and 14 normal lung samples; GSE30219 contained 181 lung cancer samples; Using GEO2R of PubMed (http://www.ncbi.nlm.nih.gov/geo/geo2r/), we analyzed the expression profiles of *EphB1* in normal lung samples, non-metastasis lung cancer samples and metastasis lung cancer samples. Overall survival was measured using the Kaplan-Meier method, and the log-rank test was used for comparison between low EphB1 expression group and high EphB1 expression group.

Oncomine (http://www.omcomine.org) data analysis was performed as previously described 16. Briefly, we evaluated *EphB1* expression in lung cancer tissues compared with corresponding normal tissues^17^-^19^ using the following threshold values: P value of 0.05, fold-change of 2. The public TCGA samples were analysed by the UALCAN database (http://ualcan.path.uab.edu/index.html). The Smad2 motif predicted from JASPAR matrix models (http://jaspar.genereg.net/).

### In vitro cell proliferation assessment

The proliferation of lung cancer cells was measured using the CCK-8 assay (Bimake, China). The cell suspension was inoculated in a 96-well plate. After treatment, 10 μl of CCK-8 solution was added to each well and the plate was incubated for an additional 4 hrs. Next, the absorbance measured at 450 nm using a microplate reader. The experiment was repeated three times, and six parallel samples were measured each time.

### Chromatin immunoprecipitation (ChIP)

ChIP assays were performed as described^20^. Briefly, A549 cells were crosslinked in 1% formaldehyde for 10 min at 37 °C to generate DNA-protein complex. Cell lysates were then sonicated and immunoprecipitated with anti-Smad2 or with IgG (control). The precipitated DNA fragments were purified and analyzed by PCR and agarose gel electrophoresis. PCR was performed using promoter-specific primers for EphB1 with amplification of the Smad2-binding regions. Primers were synthesized as follows: Forward: CCTTCCCACCCACACTGAAG; Reverse: GGTTGCCTTTGGTGTTCACTT.

### Statistical Analysis

Data are presented as the mean ± S.D. from at least three separate experiments. Statistical analyses were performed using GraphPad Prism 5 (GraphPad Software, Inc., CA, USA). Multiple group comparisons were performed using ANOVA with a post hoc test for the subsequent individual group comparisons. A p value of less than 0.05 was considered to be significant. The survival of tumour-bearing mice was analysed by Kaplan-Meier. A p value of less than 0.05 was considered to be significant.

## Results

### EphB1 expression is correlated with poor patient survival in lung cancer

**Figure 1:**
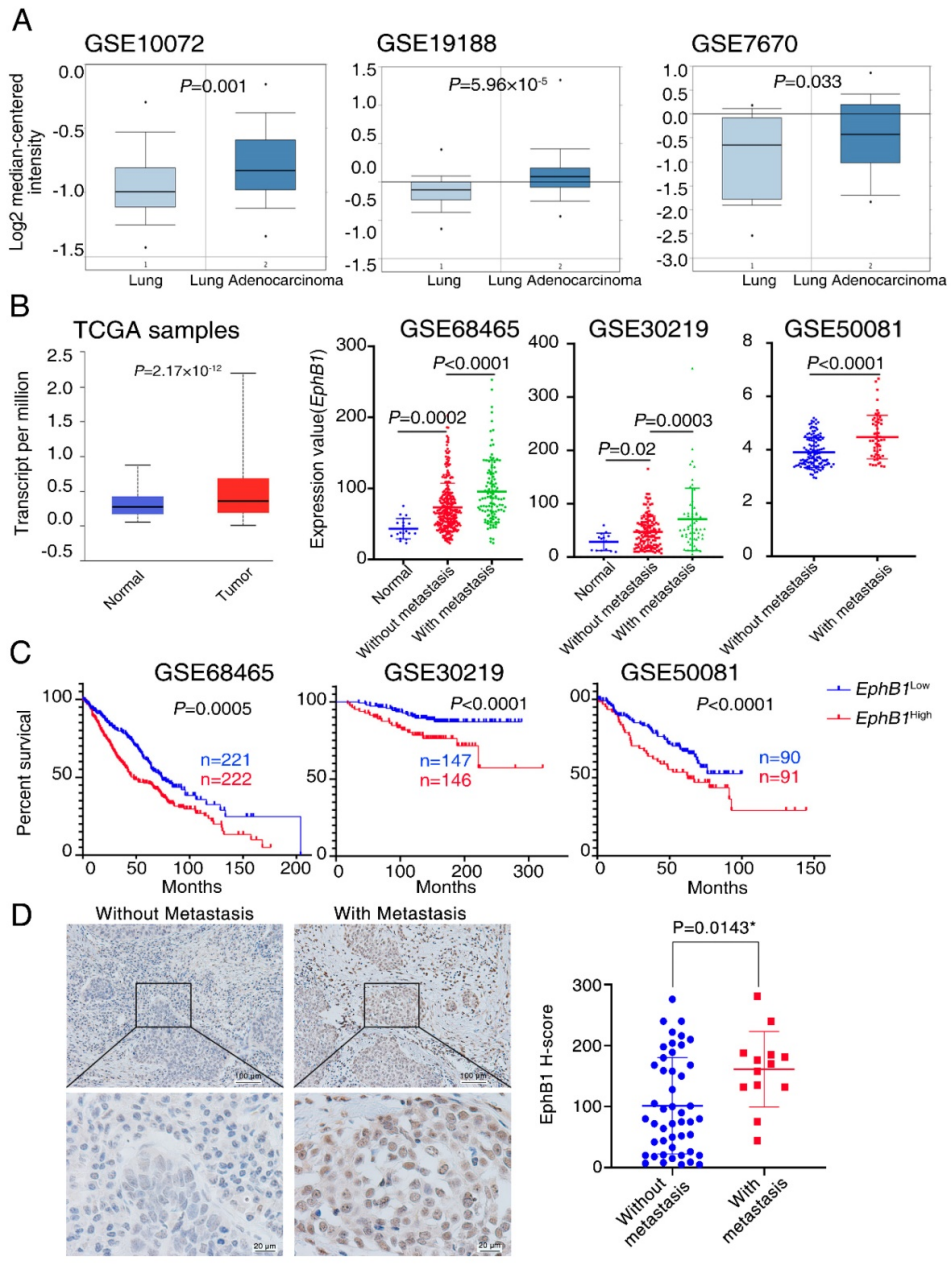
EphB1 expression is correlated with poor patient survival in lung cancer. The expression of EphB1 was analyzed in patients with NSCLC. (A) EphB1 expression in NSCLC samples and non-cancer controls; the publicly accessible gene expression data of EphB1 was obtained from Gene Expression Ominibus (GEO) database (GSE10072, GSE19188, GSE7670) and The Cancer Genome Atlas (TCGA). (B) EphB1 expression in NSCLC patients with or without metastasis and normal controls or tumor samples; The publicly accessible gene expression data of EphB1 was obtained from Gene Expression Ominibus (GEO) database (GSE68465, GSE30219, GSE50081) and The Cancer Genome Atlas (TCGA). Data were analyzed with Student's t-test, p values were shown. (C) Kaplan-Meier overall survival curves according to EphB1 expression in patient cohorts in GEO datasets. The percentage of survival patients in high EphB1 and low EphB1 groups at different time points are presented. p values were shown. (D) EphB1 expression in lung cancer patient with or without metastasis was measured by immunohistochemical staining. Scale bar: Above=100 µm; Below=20 µm; Data were analyzed with Student's t-test, *p=0.0143.

To investigate the relationship between EphB1 and lung cancer, we analyzed EphB1 expression in lung samples from cancer patients. Publicly accessible gene expression data of EphB1 was obtained from Gene Expression Ominibus (GEO) database (GSE10072, GSE19188, GSE7670, GSE68465, GSE30219, GSE50081) and The Cancer Genome Atlas (TCGA) database. EphB1 expression was significantly higher in NSCLC samples compared to non-cancer controls (Figure 1A, Figure 1B). Significant higher expression of EphB1 in cancer biopsies were found in patients with metastasis compared to non-metastatic patients with NSCLC (Figure 1B). Gene expression data for NSCLC patients was used to analysis the correlation of EphB1 and overall survival (OS). Patients with higher levels of EphB1 expression showed shorter OS compared with the patients with lower levels of EphB1 (p<0.001) (Figure 1C). EphB1 expression in lung biopsies was correlated with poor patient survival in lung cancer (Figure 1B). We verified EphB1 expression in patients by recruiting 60 NSCLC patients with or without metastasis. Clinicopathological characteristics of these patients are presented in Table 1. Consistent with results obtained from public database, the higher EphB1 expression was detected in metastatic lung cancer samples than in non-metastatic lung cancer samples (Figure 1D).

### Ligand-independent EphB1 signaling promotes cancer cell migration and invasion

**Figure 2:**
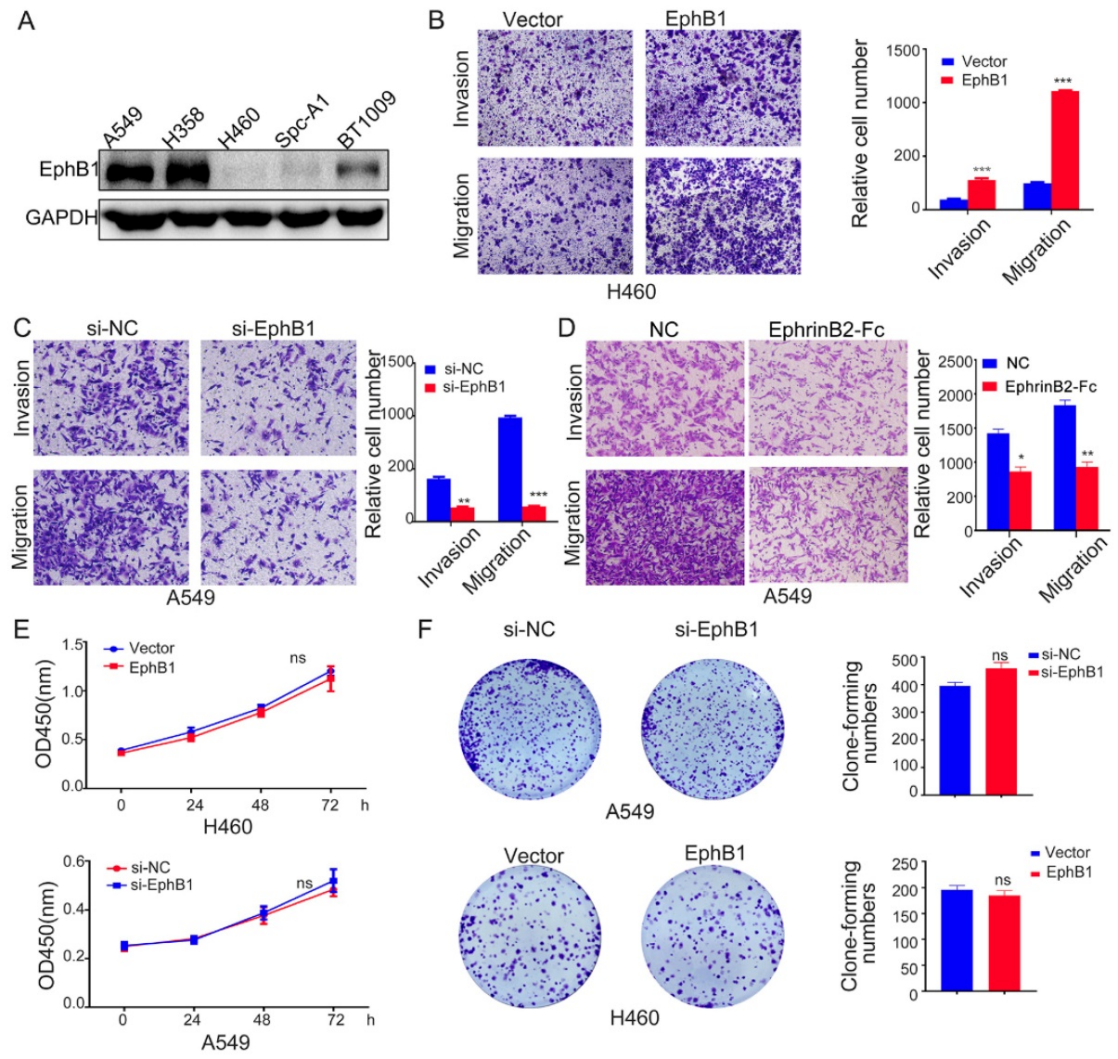
Ligand-independent EphB1 signaling promotes cancer cell migration and invasion. (A) EphB1 expression in NSCLC cell lines. (B) (C) Transwell assay was conducted to test the effect of EphB1 forced expression and (D) Ligand EphrinB2-Fc treatment on the migration and invasion of NSCLC cells. Number of cells were counted and shown in the column graph on the right of the corresponding pictures. Data are mean ± SD of three independent experiments. * P < 0.05, ** P < 0.01. (E) The proliferation of NSCLC was measured by CCK-8 or (F) Clonogenic assay after expression of EphB1 or knockdown of EphB1. Number of cell clones were counted and shown in the column graph on the right side. Data are mean ± SD of three independent experiments. ns: no significance.

To investigate the roles of EphB1 in the migration and mobility of lung cancer cell, we transfected EphB1 expression vector into H460 cells or EphB1 siRNA into A549 cells. The transwell assay revealed that EphB1 promoted the migration and invasion of lung cancer cells and knockdown of EphB1 resulted in reduced migration and invasion in A549 cells (Figure 2A, 2B and 2C). However, the ligand EphrinB2-Fc treatment on the contrary reduced migration and invasion of lung cancer cells (Figure 2D). The overexpression of EphB1 did not affect the lung cancer cell growth (Figure 2E, 2F).

### Ligand-dependent EphB1 signaling inhibits cancer cell migration and invasion through inducing the phosphorylation of EphB1

**Figure 3:**
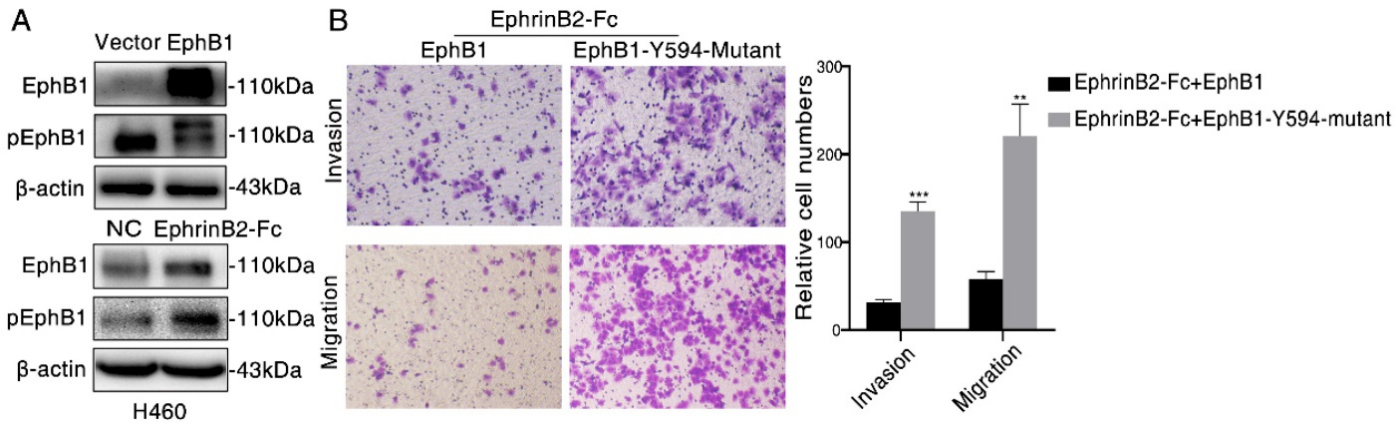
Ligand-dependent EphB1 signaling inhibits cancer cell migration and invasion through inducing the phosphorylation of EphB1. (A) Western blot was conducted to test the phosphorylation of EphB1 after transfection of EphB1 or treatment of EphrinB2-Fc; (B) Transwell assay was conducted to test the effect of EphB1 wt and mutant upon treatment of ligand EphrinB2-Fc on the migration and invasion of NSCLC cells. Number of cells were counted and shown in the column graph on the right of the corresponding pictures. Data are mean ± SD of three independent experiments. ** P < 0.01, *** P < 0.001, ns: no significance.

As the phosphorylation of EphB1 mediated by its Tyr-594 is crucial to cell migration, we then examined the effect of EphB1 forced expression and ligand EphrinB2-Fc treatment on the phosphorylation of Tyr-594. We found that transfection of EphB1 inhibited EphB1 Tyr-594 phosphorylation, while treatment of EphrinB2-Fc promoted EphB1 Tyr-594 phosphorylation (Figure 3A). It suggests the forced overexpression of EphB1 inhibits EphB1 forward signaling and exogenous EphrinB2-Fc promotes EphB1 forward signaling.

In order to investigate if the activation of EphB1 forward signaling affect the cell mobility, we transfected wild type EphB1 or EphB1 Y594 mutant into A549 cells. The exogeneous treatment of EphrinB2-Fc obviously inhibited the migration and invasion of EphB1 wt transfected cells, but significantly improved the migration and invasion of EphB1 Y594 mutant transfected cells (Figure 3B). It demonstrated that phosphorylation of EphB1 reduces migration and invasion of lung cancer cells, whereas the ligand-independent EphB1 promotes migration and invasion of lung cancer cells.

### Ligand-independent EphB1 mediates TGF-β-activated CDH2

**Figure 4:**
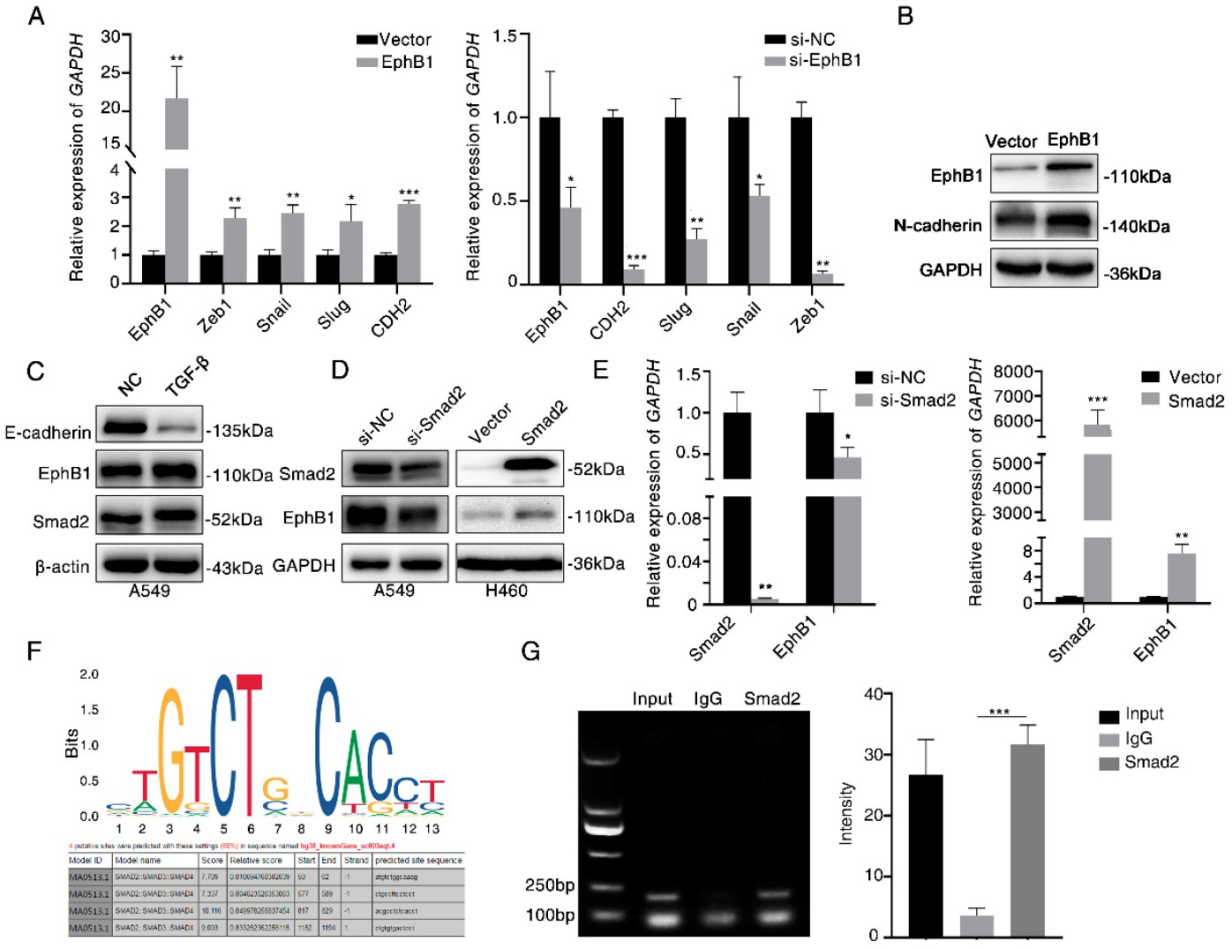
Ligand-independent EphB1 mediates TGF-β-activated N-cadherin. (A) Expression of mesenchymal molecules in lung cancer cells after transfection of EphB1 or knockdown of EphB1 quantified by real-time PCR. *p<0.05; **p<0.01; ***p<0.001. (B) Expression of CDH2 upon transfection of EphB1 measured by Western blot. (C) Expression of EphB1 upon treatment of TGF-β measured by Western blot. (D) Western blot was conducted to test the expression EphB1 after transfection of Smad2 or knockdown of Smad2. (E) Expression of EphB1 after transfection of Smad2 or knockdown of Smad2 quantified by real-time PCR. (F) The putative Smad2/3 binding sites in EphB1 promoter. (G) ChIP assay in 293 cells to detect the binding of Smad2 to the promoter of EphB1. Mean values± SD of three independent experiments are shown on the right side. IgG indicates nonspecific Ab.

To investigate the mechanism of how EphB1 overexpression promotes the migration and invasion of lung cancer cells, we compared the expression of EMT related molecules between cells with or without forced expression of EphB1. We found that the transfection of EphB1 promoted the expressions of mesenchymal molecules such as *Snail*, *Slug*, *CDH2*, *Zeb1*, whereas knockdown the expression of EphB1 inhibited the expressions of mesenchymal molecules (Figure 4A). Western blot was performed to confirm the upregulation of *CDH2* induced by *EphB1* overexpression (Figure 4B).

TGF-β signaling is the main regulator in cell migration and invasion. We then examined if TGF-β regulates EphB1 expression. We found that TGF-β enhanced the expression of EphB1 (Figure 4C). Transfection of Smad2 enhanced the expression of EphB1 (Figure 4D, 4E). We then performed a ChIP assay to elucidate if TGF-β-activated Smad2 can be recruited to *EphB1* promoter. The putative Smad2/3 binding sites were shown in Figure 4F. ChIP assay performed on Smad2-transfected HEK293 cells revealed that Smad2 bound to *EphB1* promoters (Figure 4G).

## Discussion

Here, we studied the ligand mediated trans-forward EphB1 signaling and ligand-independent cis-attenuation signaling contribute to the migration and invasion of lung cancer cells. TGF-β-activated Smad2 directly binds to *EphB1* promoter and transcriptionally modulates *EphB1* expression.

Eph family of receptor tyrosine kinases and their ligands mediate many physiologic and pathologic effects by a multiple signaling mode^21^. Ephrins and Eph tyrosine kinases initiate a unique bidirectional signal (forward and reverse signals) on both the receptor-expressing and ligand-expressing cells^22^,^23^. Furthermore, Eph receptors and ligand ephrins make their cis-interaction when the ligand and receptor are expressed in the same cells, while they interact in trans when ligand and receptor are expressed in different cells^21^. *Cis* interactions may be one of the strategies that adopted by cancer cells to escape the tumor suppressive effects of Eph receptor signaling induced by ephrins binding in *trans*^24^-^26^. Eph receptors and ephrins coexpressed in the same cells can attenuate receptor activation in trans by hindering the binding of ephrins and Eph receptors in trans^27^. The coexpression of Ephrin-A3 can block the ability of EphA2 and EphA3 to link ephrins in *trans* and become activated, while Ephrin-B2 can deter not only EphB4 but also EphA3 in the cancer cells^27^. Eph receptors are often upregulated in many types of cancer, although decreased Eph receptor levels were also been observed in certain types of cancer^28^. In contrast to the overexpression of Eph receptors in cancer, Eph receptor forward signaling that triggered by tyrosine phosphorylation inhibits tumor cell growth^29^,^30^. Higher expression of Ephrin-B2 is correlated with poor overall survival and disease-free survival in head and neck squamous cell carcinoma, pancreatic adenocarcinoma and bladder urothelial carcinoma^31^. The paradoxical functions of Eph are influenced by the cis- or trans- signaling. In this study, EphrinB2-Fc provided in trans mode can elicit EphB1 forward signaling, leading to reduced mobility and invasion. The overexpression of EphB1 without exogenous stimulation of Ephrin-B2, however, promotes mobility and invasion through upregulating EMT molecules.

TGF-β signaling is thought to drive EMT and trigger apoptosis. TGF-β-activated Smad3/4 directly binds to *CDH2* promoter and transcriptionally regulates* CDH2* in NSCLC^32^. Our study found that Smad2 binds to *EphB1* promoter and transcriptionally regulates *EphB1* expression. The endogenous expression of EphB1 promotes migration and invasion of NSCLC through upregulating *CDH2*. It demonstrated that TGF-β regulates CDH2 by directly binding to *CDH2* promoter or indirectly through transcriptionally regulating *EphB1*.

## Conclusion

In conclusion, the roles of EphB1 in cancer cell invasion and migration are context-dependent and involve the cis- or trans- interactions between receptor and ligands. EphB1 is transcriptionally regulated by Smad2 and mediates TGF-β signaling in a ligand-independent manner.

## Figures and Tables

**Table 1:** Clinical characteristics and EphB1 expression in 60 cases of NSCLC.

Characteristics	n	EphB1 expression		P -value
		Low or none no. case (%)	High no. case (%)	
Total	60	29	31	
Gender				
Female	29	16	13	0.305
Male	31	13	18	
Age				
≤60	30	13	17	0.438
>60	30	16	14	
Distant metastasis				
Negative	47	27	20	**0.007**
Positive	13	2	11	

**Table 2:** Primer sequence for real-time PCR

Gene	Forward primer (5′- to 3′)	Reverse primer (5′- to 3′)
*EphB1*	ATGCGCTTCACTGTGAGAGAC	ATTCCGAGTAAGAGGCCCAAA
*CDH2*	AGCCAACCTTAACTGAGGAGT	GGCAAGTTGATTGGAGGGATG
*Snail*	TCAAGATGCACATCCGAAGCC	TTGTGGAGCAGGGACATTCG
*Zeb1*	GCACAACCAAGTGCAGAAGA	GCCTGGTTCAGGAGAAGATG
*Slug*	TGGTCAAGAAACATTTCAACGCC	GGTGAGGATCTCTGGTTTTGGTA
*Smad2*	CCGACACACCGAGATCCTAAC	GAGGTGGCGTTTCTGGAATATAA
*CDH1*	TGAAGCCCCCATCTTTGTGC	GGCTGTGTACGTGCTGTTCT
*GAPDH*	AACGGATTTGGTCGTATTGG	TTGATTTTGGAGGGATCTCG

## Abbreviations

NSCLC: Non-Small-Cell Lung Cancer
EMT: Epithelial-mesenchymal transition
